# CRISPR/Cas9: A Practical Approach in Date Palm Genome Editing

**DOI:** 10.3389/fpls.2017.01469

**Published:** 2017-08-23

**Authors:** Muhammad N. Sattar, Zafar Iqbal, Muhammad N. Tahir, Muhammad S. Shahid, Muhammad Khurshid, Abdullatif A. Al-Khateeb, Suliman A. Al-Khateeb

**Affiliations:** ^1^Department of Environment and Natural Resources, Faculty of Agriculture and Food Sciences, King Faisal University Al-Ahsa, Saudi Arabia; ^2^Akhuwat-Faisalabad Institute of Research, Science and Technology Faisalabad, Pakistan; ^3^National Institute for Biotechnology and Genetic Engineering Faisalabad, Pakistan; ^4^Department of Crop Sciences, College of Agricultural and Marine Sciences, Sultan Qaboos University Al-Khoud, Oman; ^5^Institute of Biochemistry and Biotechnology, University of the Punjab Lahore, Pakistan; ^6^Plant Biotechnology Department, Faculty of Agricultural and Food Sciences, King Faisal University Al-Ahsa, Saudi Arabia; ^7^Ministry of Environment, Water and Agriculture Riyadh, Saudi Arabia

**Keywords:** CRISPR/Cas9, date palm, genome editing, multiplexing, loss of and gain-of-functions

## Abstract

The genetic modifications through breeding of crop plants have long been used to improve the yield and quality. However, precise genome editing (GE) could be a very useful supplementary tool for improvement of crop plants by targeted genome modifications. Various GE techniques including ZFNs (zinc finger nucleases), TALENs (transcription activator-like effector nucleases), and most recently clustered regularly interspaced short palindromic repeats (CRISPR)/Cas9 (CRISPR-associated protein 9)-based approaches have been successfully employed for various crop plants including fruit trees. CRISPR/Cas9-based approaches hold great potential in GE due to their simplicity, competency, and versatility over other GE techniques. However, to the best of our knowledge no such genetic improvement has ever been developed in date palm—an important fruit crop in Oasis agriculture. The applications of CRISPR/Cas9 can be a challenging task in date palm GE due to its large and complex genome, high rate of heterozygosity and outcrossing, *in vitro* regeneration and screening of mutants, high frequency of single-nucleotide polymorphism in the genome and ultimately genetic instability. In this review, we addressed the potential application of CRISPR/Cas9-based approaches in date palm GE to improve the sustainable date palm production. The availability of the date palm whole genome sequence has made it feasible to use CRISPR/Cas9 GE approach for genetic improvement in this species. Moreover, the future prospects of GE application in date palm are also addressed in this review.

## Introduction

The erosion of plant genetic resources and the global climate changes confront us with enormous challenges including biotic and abiotic stresses (Arzani and Ashraf, 2016). Add to these limitations the fact that date palm (*Phoenix dactylifera* L.) is composed of genetically discrete clones representing thousands of cultivars without the benefits of a dynamic mutation-recombination system (Al-Khayri et al., 2015). The generation of an explosion of knowledge and technology related to genomics and genetics over the last few decades is promising in providing powerful tools for future development of higher-yielding cultivars (Arzani and Ashraf, 2016). The genome editing (GE) tools like zinc finger nucleases (ZFNs), transcription activator-like effector nucleases (TALENs), and the contemporary clustered regularly interspaced short palindromic repeats (CRISPR) along with CRISPR-associated protein 9 (Cas9) have established their hierarchy in editing plant genomes. By using these GE tools, genome modifications have been accomplished in various plants by ZFNs (Shukla et al., 2009; Townsend et al., 2009; Zhang and Voytas, 2011; Curtin et al., 2013; Pater et al., 2013; Qi et al., 2013) and TALENs (Christian et al., 2013; Sun et al., 2013; Wendt et al., 2013; Zhang et al., 2013). Recently developed CRISPR/Cas9 has gained popularity among the scientific community in a short period of time. In comparison to all these three GE tools, CRISPR/Cas9 offers several advantages over TALENs and ZFNs which include target design simplicity, improved efficacy and precision, multiplexing, least off-targets, ability to target multiple alleles, cost effective, easy delivery and execution, and availability of *in silico* techniques to design and evaluate the designed single-guide RNA (sgRNA). Additionally, in ZFNs and TALENs sequence specificity is conferred by DNA-binding domain of protein while in CRISPR/Cas9 system sgRNA mediate this, no protein engineering is involved in CRISPR system. Unlike to predecessor GE—CRISPR system can cleave methylated target sequence (reviewed by Bortesi and Fischer, 2015). Moreover, the protein domain engineering of the target DNA is a pre-requisite for multiplexing using TALEN and ZFNs, which makes these techniques less-suitable and limited for multiplexing (Lowder et al., 2017). Whereas, multiplexing in CRISPR/Cas9 system requires only multiple sgRNAs jointly expressed with the Cas9 (detailed discussion in Section “Verifications of the Genome Editing Events in Date Palm”). CRISPR/Cas9 system has been modified into a two-component system; Cas9 and an sgRNA. The Cas9 protein cleaves the target DNA by a synthetic sgRNA, which comprised of crRNA and tracrRNA. CRISPR/Cas9 has been successfully used as a GE tool to confer resistance against citrus canker (Jia et al., 2016; Peng et al., 2017), rice blast (Wang et al., 2016; Kanda et al., 2017), powdery mildew (Nekrasov et al., 2017; Zhang et al., 2017), phytophthora (Fang and Tyler, 2016), and multiple plant viruses (Iqbal et al., 2016; Khatodia et al., 2017) in the model plants as well as in the commercial crops. Moreover, it has also been successfully tested, either transiently or through stable transformation, for precise GE and knockout mutations in the woody plants citrus (Jia and Wang, 2014), populous (Fan et al., 2015; Zhou et al., 2015), and apple (Nishitani et al., 2016). Thus, programmable GE in woody fruit trees holds a substantial potential elucidating off-screen molecular mechanisms in governing flowers, fruits, and whole plant developments.

Nevertheless, no GE tool has been exploited in date palm genome engineering. Date palm (family *Palmae* or *Arecaceae*) is a high ranked diploid (2*n* = 36), monocotyledonous, dioecious, perennial woody fruit tree and has high socio-economic significance in Oasis agriculture (Mahmoudi et al., 2008). The average date palm genome is about 670 Mb in size (Al-Dous et al., 2011; Al-Mssallem et al., 2013) and comprises of 18 chromosomes. It is native to Arabian Peninsula, possibly originated from southern Iraq and has vast diversity from Mauritania to Pakistan (Pintaud et al., 2011). Other regimes where date palm is being cultivated are sub-Saharan African countries, Australia, United States (California), Peru, and some other warmer parts of the world. Currently, 450 date palm varieties are grown in Kingdom of Saudi Arabia and more than 2000 cultivars in the world. This majestic plant is known as “Tree of life” since the settlements of ancient human in the hot and barren parts of the world. Besides a nutritious source of human diet, it is a continuous source of raw material for housing, sheltering, and handicrafts in the harsh dry environments of Southwest Asia and North Africa (Jain et al., 2011). Nevertheless, over the time date palm agronomy has been shifted to monoculture instead of traditional cultivation. Such a situation brought severe genetic erosion of many productive cultivars and trembled the date palm agro-biodiversity in many areas (Jain et al., 2011). Additionally, several biotic and abiotic stresses further worsen the situation.

Previously, biotechnological approaches, such as plant tissue culture, marker-assisted breeding and DNA finger printing, have been used in date palm genomics but failed to bring a significant improvement. Additionally, conventional agronomic practices and breeding approaches in date palm are not cost effective as performing three backcrosses it usually takes 30 years of breeding. For the sustainability of date palm, employment of new techniques in date palm breeding programs is highly needed to develop tolerant varieties and enrich the existing germplasm. This can be achieved by modifying the date palm genome against various biotic and abiotic stresses by overexpressing or downregulating the key genes involved in biochemical pathways, or by engineering resistance against various pests and diseases. Additionally, the dissection of genetic information in date palm would help in understanding the role of various genes involved in sex determination, enzymatic reactions controlling fruit ripening, fruit sweetness, and fruit quality. In order to determine the universal efficacy of CRISPR/Cas9, extensive investigation in date palm is also necessary.

## Major Abiotic and Biotic Constraints In Date Palm Cultivation

During the long life span, high salinity, extreme drought regimes, and blazing heat are major abiotic stresses affecting date palm. Our understanding about the molecular mechanisms regarding abiotic stresses is still very limited, however, a proteomic analysis recently identified 47 differentially expressed proteins in salt and drought affected date palm plants (El Rabey et al., 2015). Among various biotic stresses, the bayoud disease infection (caused by *Fusarium oxysporum*) in North Africa (Sedra, 2007), red palm weevil (*Rhynchophorus ferrugineus*) in Middle East Asia and Mediterranean regions (Ferry and Gomez, 2002) are the most important (Supplementary Table 1). Furthermore, 34 diverse fungal and *Oomyces* species have been found associated with date palm root diseases (Al-Sadi et al., 2012).

**FIGURE 1:**
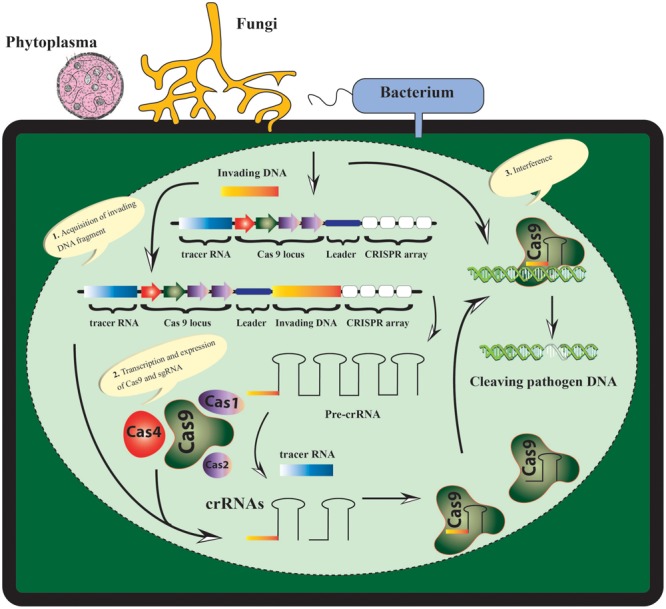
The CRISPR/Cas9-based resistance model in date palm depicting the recognition and disruption of the pathogen genetic material in three steps: acquisition, expression, and interference). During acquisition the invading DNA is integrated and duplicated into the CRISPR-locus at the leader side. The expression step involves the active transcription and expression of pre-CRISPR RNA (Pre-crRNA), which is further processed into mature crRNAs specifically with the help of different Cas proteins. During the third step of interference, any complementary target region of the foreign genetic material is recognized and cleaved as guided by crRNA and Cas9 protein.

The mounting climatic and population scenario, long date palm breeding time along with biotic factors have left no choice for the scientists except development of genetic resistance for sustainability of date palm. However, developing a high yielding, resistant and good fruit quality cultivar demands a stringent, comprehensive and reliable methodology. The CRISPR/Cas9-based approaches can be harnessed to target the genomes of date palm’s pathogens directly or indirectly. The date palm insect/pests can be targeted by CRISPR/Cas9-based approaches through “gene drives” to circumvent their population (**Figure 1**).

## Limitations In Date Palm Functional Genomics

Despite of a rich agricultural history and economic importance of date palm, the application of high-throughput technologies started during last decade. The palm family has been an ignored group to understand their developmental potential for genetic improvement (Bekheet and Hanafy, 2011). The first genetic map of the date palm “cultivar Khalas” has been constructed quite recently (Mathew et al., 2015), soon after sequencing of chloroplast (Khan et al., 2005; Yang et al., 2010; Sabir et al., 2014), mitochondria (Fang et al., 2012), and the whole nuclear genome (Al-Dous et al., 2011; Al-Mssallem et al., 2013). Furthermore, the molecular variations in the date palm genome have also been cataloged recently (Hazzouri et al., 2015). The whole genome sequencing of date palm also facilitated other relative studies including transcriptomic analysis (Yin et al., 2012), miRNAs expression profiling (Xiao et al., 2013; Xin et al., 2015; Yaish et al., 2015), comparative analysis of *P. dactylifera* and oil palm fruits (Bourgis et al., 2011), construction of genetic models (Zhang et al., 2011), and genetic diversity using single-nucleotide polymorphism (SNPs) data (Sabir et al., 2014; Mathew et al., 2015). Very recently, the applications of -omics coupled with bioinformatics were employed to determine and characterize simple sequence repeats (SSRs) for the endowment of SSR database in date palm genome (Zhao et al., 2012; Mokhtar et al., 2016). Moreover, same approaches have also been employed recently for *in silico* characterization and molecular structuring of *DnMRE11*, a gene involved in double stranded DNA-breaks repair, in date palm cultivar “Deglet Noor” (Stracker and Petrini, 2011; Rekik et al., 2015). A miRNA profiling of the date palm cultivar “Khalas” revealed 153 conserved homologs, 89 variants, whereas 180 novel miRNAs directly involved in the salt adaptation (Yaish et al., 2015). An *in silico* analysis by the same researchers unraveled that these miRNAs could directly regulate many salt tolerance related genes in leaves and roots, respectively. Similarly, computational studies of miRNA in date palm revealed their involvement in fruit development (Xin et al., 2015) and evolution (Xiao et al., 2013). Besides, the recent gene annotation in date palm no experimental validations are still available for their expression and biological functions in this species (Bourgis et al., 2011; Zhang et al., 2012). Therefore, predicting the expression of a relevant gene in date palm is not as authenticated as its ortholog in other plant species. Likewise, most of the available information regarding date palm genome is from a single cultivar “Khalas,” which may or may not match with the cultivar used in other studies. Thus, exploring the intrinsic molecular mechanisms governing certain gene functions and transcriptional regulation in date palm is vital for its sustainable production across the globe. Although, genetic diversity in different date palm cultivar can be assessed through NGS and SNPs techniques (Yang et al., 2010; Fang et al., 2012; Khan et al., 2012; Sabir et al., 2014) but studying functional genomics in date palm is very tedious due to longer vegetative regimes, low efficacy of genetic transformation, and limited survival of mutants. The evasion of genetic barriers in the genetic improvement of such tree plants is possible through the application of genetic transformation and GE tools.

## A Generalized Stepwise and Basic Strategy for CRISPR/Cas9 Implications In Date Palm

**FIGURE 2:**
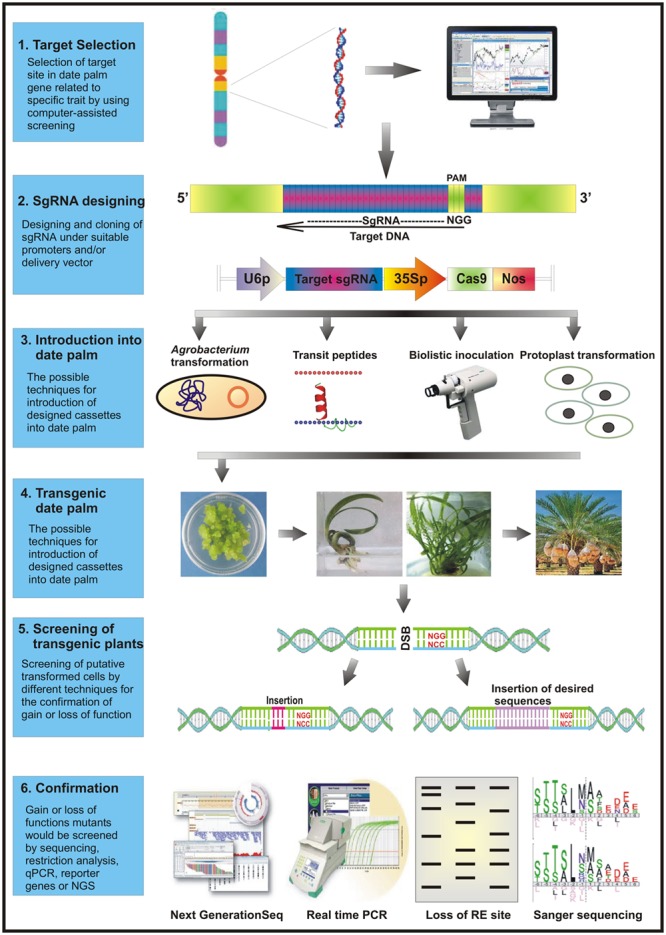
A schematic diagram representing the execution of CRISPR/Cas9 based system in date palm genome editing. The most vulnerable target sites in the desired gene(s) are selected specifically using online available web sourcing to design primers for complementary 20-nucleotides. The target specific sgRNA and Cas9 cassettes are constructed either in a single binary vector or separate expression vectors. These cassettes are then co-transformed *in vivo* into the plant cells employing a suitable transformation method. Following the putative transformation, the mutated cells are screened an analyzed for target-specific mutations using reporter genes, endonucleases, polyacrylamide gel electrophoreses, or high throughput sequencing techniques. The successfully transformed cells are then selected for further downstream applications and analysis.

A stepwise methodology for successful execution of CRISPR/Cas9 system in date palm is outlined in the succeeding section and the **Figure 2**.

### Data Mining and Target Selection (sgRNA Design)

The primary and the most important step in GE is the selection of a target region in the genome. The major challenges during the selection of a target region in date palm may include genome polymorphism, off-targets (discussed in section “Countering off-targets in date palm genome editing”), presence of introns (that may get further aggravated by alternative splicing) and presence of SNPs. However, many tools are available to deal with such problems and generally categorized into three major steps: (i) selection of target region or sites/sgRNA designing, (ii) verification of the designed sgRNA for possible off-targets, and (iii) evaluation of on- and off-target cleavage rates (Lee et al., 2016). To ascertain successful date palm genome engineering, several available web-based bioinformatics and data mining tools (like CCTop, ATUM, MIT CRISPR design, Alt-R^TM^ CRISPR-Cas9 System, CHOPCHOP, CROP-IT, GT-Scan, sgRNA Designer, Cas-OFFinder, etc.) can impart very promising role.

### Selection of CRISPR/Cas9 System

Successful execution of CRISPR/Cas9 system in date palm requires expression of bacterial Cas9 protein into date palm cells. Codon optimization and selection of suitable promoter(s) is a pre-requisite to accomplish CRISPR/Cas9 system in plants (Belhaj et al., 2013). Several plant promoters (CaMV 35s, CMV, EF1A, LTR, and UBO) have been implicated to drive Cas9 expression. In many studies, plant RNAIII promoters, like U3 and U6, have also been used successfully (Belhaj et al., 2013).

Another important consideration is the selection of efficient Cas9. Generally, eukaryotic optimized *SpCas9* have efficient GE ability and used vastly in eukaryotes. Nevertheless, many studies have used plant codon-optimized version of Cas9 (Jiang et al., 2013; Li et al., 2013; Miao et al., 2013; Shan et al., 2013) with improved efficacy. Beside these versions of Cas9, a dCas9 version lacking nuclease ability has been developed to employ this tool as gene silencing [CRISPR interference (CRISPRi)] and gene activation [CRISPR activation (CRISPRa)] rather than GE tool (Gilbert et al., 2013; Lei et al., 2013). Such modifications will further widen the applications of CRISPR/Cas9 system and can potentially be opted for date palm genome modifications and regulations.

### CRISPR/Cas9 Cassette Delivery into Date Palm

Next step is the assembly of whole cassette (carrying sgRNA, CRISPR, Cas9 and, if needed, a nuclear localization signal) into any suitable plant based expression vector to deliver the assembled construct into date palm genome. Successful delivery of the system into date palm with high precision will be a challenging task. To deliver CRISPR/Cas9 constructs into date palm tissues various methods such as protoplast transformation, polyethylene glycol-mediated transformation, biolistic inoculations, transit peptides (Lee et al., 2008), or some plant virus-based vectors can be used (Butler et al., 2016). However, stable genetic transformation in date palm through particle bombardment (Mousavi et al., 2014) and/or Agrobacterium-mediated genetic transformation of callus, embryos and immature tissues is the most common and successful method for effective applications of CRISPR/Cas9 in plants (Ma et al., 2016). Direct transformation of date palm genome can be tricky therefore, adapting a DNA-free strategy to transfect date palm protoplast by the preassembled cassettes would be a good choice. Such strategy is highly useful for the vegetatively propagated perennial plant species (Woo et al., 2015).

### Regeneration and Screening of Targeted Mutations in Date Palm

After successful transfection, the date palm plantlets can be regenerated on selection media and subjected to screening of gene editing events by employing different strategies (**Figure 2**). The phenotypic and genotypic screening requires detection and confirmation of the targeted mutation (Hua et al., 2017).

### Verifications of the Genome Editing Events in Date Palm

Screening and confirmation of specific gene edited mutants induced by CRISPR system is not only crucial but important too for downstream processing.

A quick way to verify the efficacy of GE is the use of reporter genes (such as GFP, RFP, YFP, and GUS) as a marker of editing events. To use this system, the reporter gene should bear a frame shifting at target site or alternatively contain duplicated region that could be corrected by CRISPR/Cas9 system after GE (Siebert and Puchta, 2002; Jiang et al., 2013; Mao et al., 2013; Feng et al., 2014). Alternatively, an internal or introduced endonuclease site can be targeted during Cas9/sgRNA cleavage (Jiang et al., 2013; Shan et al., 2013; Xie and Yang, 2013). The perturbed endonuclease site can be confirmed through PCR amplification and ultimately a successful GE will be ensured.

The successful GE events through CRISPR/Cas9 system can also be confirmed by using polyacrylamide gel electrophoresis (PAGE). Single-stranded DNAs with nucleotide variations can exhibit different migration rate due to change in the DNA conformations. This technique referred as single-stranded conformation polymorphism and could be used to detect targeted mutations induced by CRISPR/Cas9 (Zhang Y. et al., 2016). High-resolution melting assay offers another powerful tool for GE verifications, where mutation is determined in PCR amplicons on the basis of difference in their melting temperature. Though, sensitivity is low and sequence of mutated target gene cannot be determined but can be practiced for a preliminary screening of mutations induced by CRISPR/Cas9 system (Dahlem et al., 2012; Fauser et al., 2014).

Next generation or high-throughput sequencing of the PCR amplicons or whole genome is highly sensitive and efficacious method of detecting the mutation in the target region. Although, this method is expensive and time consuming but is highly reliable and robust to detect low frequency mutations and off-target mutations in the whole genome (Fauser et al., 2014; Feng et al., 2014). Using old fashioned PCR amplification, subsequent cloning and sequencing of the targeted gene through Sanger sequencing platform can provide relatively inexpensive and efficient method for the confirmation of mutations at the target site. This approach is convenient for determining either simple mutations or complicated chimeric mutations (Zhou et al., 2014; Ma et al., 2015). A real-time quantitative PCR can enable the measurement of transcript levels in both coding and non-coding targets. These tools greatly facilitate the analysis of targeted sites.

### Gene Stacking Using Multiplex CRISPR/Cas9 Model in Date Palm

**FIGURE 3:**
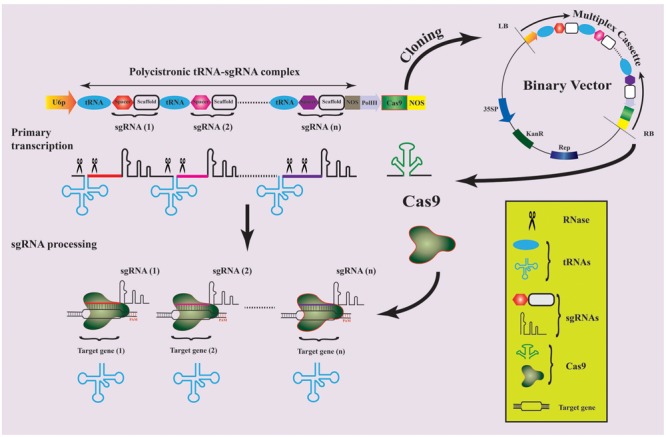
Schematic presentation of multiplex genome editing strategy in date palm. A preferred assemblage of multiplex cassette is shown for different sgRNAs to target different genes simultaneously. The spacers and sgRNA scaffold can be inserted between adjacent tRNAs followed by a NOS terminator at the end. The second cassette can be expressed from Pol-III promotor and NOS terminator sequences sharing the same binary vector with sgRNA cassette. The whole multiplex cassette can then be transcribed and expressed separately in the date palm genome to carry out genome editing.

Precise modification of multiple genes, involved in controlling a particular trait, in one go have been a long standing interest of genome scientists. Recently, a powerful system for multiplex genome engineering has been employed in rice, where hijacking of endogenous tRNA-processing mechanism was achieved to generate multiple sgRNAs from a single construct (Xie et al., 2015). In this system, constructs are designed in polycistronic tRNA–sgRNA (PTG) form with repeating units of target specific spacer and sgRNA scaffolds, which can be separated by conserved tRNA for multiplexing (**Figure 3**). During transcription, the endogenous RNases will cleave the PTG leaving multiple sgRNAs that direct the Cas9 to respective target sites. The tRNAs will also lead to sgRNA over expression by increasing the Pol III transcriptions. By using this method, 3–31 times increased level of GE with 15–19% higher mutation have been achieved when compared to other CRISPR/Cas9-based multiplexing approaches (Xie et al., 2015).

PTG-based multiplex CRISPR/Cas9 system can be used for date palm GE because of its certain advantages over other multiplexing techniques, which include use of short 5′ spacer sequences prior to sgRNAs, editing/deletion of non-coding regions (Xie et al., 2015) and efficient production of multiple sgRNAs from one construct using endogenous RNA-processing machinery with improved multiplex GE abilities (Xie et al., 2015; Ding et al., 2016).

The successful implications of PTG-based method include GE of allopolyploid wheat (Wang et al., 2016) (**Table 1**) and *Zea mays* where enhanced mutagenesis efficiency was observed (Qi et al., 2016). These implications are not limited to plants but quite recently, three human genes related to *histone deacetylase* (HDAC) have been targeted successfully (Dong et al., 2017). By utilizing the universal tRNA-based approach, successful manipulation of multiple genomic loci in date palm can potentially be achieved against different biotic and abiotic challenges (Supplementary Table 1 and **Figure 3**) particularly red palm weevil and bayoud disease. Comparative genomic studies of date palm deciphered that more than 50% date palm predicted ORFs matches with rice ORFs (Al-Dous et al., 2011) while, proteome comparison showed that ∼8000 gene families of date palm are common to both monocot (rice and sorghum) and dicot plants (*Arabidopsis thaliana* and grapevine) (Al-Mssallem et al., 2013). Thus, different date palm genes can be targeted by designing the sgRNAs referring to the successful multiplex GE in various crops (**Table 1**).

**Table 1:** Examples of different plant species (genes) multiplexed using CRISPR/Cas9 GE approach.

Organism	Target(s)/gene(s)	Description of gene(s)	Cas9 promoter	sg Promoter	Binary vector/backbone	Reference
**Dicotyledonous plants**						
*Arabidopsis thaliana*	GLV family	GOLVEN gene family regulating root stem cells	*Arabidopsis* ubiquitin 10 promoter	*Arabidopsis* U6 promoter	pCUT binary vector	Peterson et al., 2016
	PYR1, PYL1, PYL2, PYL4, PYL5, PYL8	PYR/PYL gene family	pAtUBQ1 promoter	AtU6-26, AtU3b, and At7SL-2 promoters	pEx-6XsgR-PYL114285-Cas9 binary vector	Zhang Z. et al., 2016
	AtPDS3	Phytoene desaturase; photobleached phenotype	Constitutive 35SPPDK	*Arabidopsis* U6 polymerase III promoter	pFGC-RCS binary vector	Li et al., 2013
	AtRACK1b, AtRACK1c	Receptor for activated C kinase 1 (RACK1) family				
	CHLI1, CHL12	Magnesium-chelatase subunit I (CHLI); pale green to albino plants	AtUBQ1 promoter	AtU6 promoter	pCAMBIA1300	Mao et al., 2013
	TT4	TRANSPARENT TESTA 4				
	RTEL1	Regulator of telomere length 1, AT1G79950	Ubiquitin 4-2 promoter from *Petroselinum crispum* (PcUbi4-2)	*Arabidopsis* U6-26 promoter (AtU6-26)	Binary vectors was derived from pPZP201; pDe-Cas9-D10A	Schiml et al., 2014
	At5g55580	Mitochondrial transcription termination factor (mTERF)	Maize ubiquitin promoter (P_ubi_) or the cauliflower mosaic virus 35S promoter (P_35S_)	U3 and U6 small nuclear RNA promoters from *Arabidopsis*; AtU3b, AtU3d, AtU6-1, AtU6-29	pYLCRISPR/Cas9 binary vectors based on the pCAMBIA1300 backbone	Ma et al., 2015
Tomato (*Solanum lycopersicum*)	SlyGABA-TP1, SlyGABA-TP2, SlyGABA-TP3, SlyCAT9, and SlySSADH	γ-Aminobutyric acid (GABA) metabolic pathway	Ubiquitin promotor	LacZ-AtU3d, AtU3d, AtU3b, AtU3b, AtU6-1 or AtU6-29	pYLCRISPR/Cas9	Li et al., 2017
	*SlAGO7*	*ARGONAUTE7* (*SlAGO7*) for post-transcriptional silencing of *AUXIN RESPONSE FACTOR*	CaMV 35S promoter	*A. thaliana* U6 promoter	pAGM4723	Brooks et al., 2014
	*Solyc08g041770, Solyc07g021170, Solyc12g044760*	Three homologs of *Solyc11g064850* control tomato reproductive development				
*Nicotiana benthamiana*	*PDS*	*Phytoene desaturase* gene related with albino leaf phenotype	CaMV 35S promoter	CaMV 35S	pBI121	Upadhyay et al., 2013
	*PDS*	Albino leaf phenotype	CaMV 35S promoter	*Pea early browning virus* (PEBV) promoter	pK2GW7, TRV2 RNA2 vector	Ali et al., 2015
	*PCNA*	*Proliferating cell nuclear antigen* gene related with DNA replication				
*Nicotiana tabacum*	*XylTA, XylTB*	*β(1,2)-Xylosyltransferase (XylT)*	35SPPDK	U6 promoter	pFGC-pcoCas9	Mercx et al., 2017
	*FucTA, FucTB, FucTC, FucTD*	*α(1,3)-Fucosyltransferase (FucT)* plant specific glycans				
	*NtPDS*	Albino leaf phenotype	2 × CaMV 35S promoter	*Arabidopsis* U6-26 promoter	pORE O4	Gao et al., 2015
	*NtPDR6*	Multiple branches				
Soybean (*Glycine max*)	*Glyma01g38150*; *Glyma11g07220*; *Glyma04g36150*; and *Glyma06g18790*	*A. thaliana DDM1 and MET1* orthologs	2 × CaMV 35S promoter	*Medicago truncatula* U6.6 polymerase III promoter	p201N Cas9	Jacobs et al., 2015
**Monocotyledonous plants**						
Rice (*Oryza sativa*)	DEP1, EP3, Gn1a, GS3, GW2	Panicle architecture and yield related genes	2× 35S promoter	U6 promoter	pC1300-Cas9 binary vector	Shen et al., 2017
	BADH2	*Betaine aldehyde dehydrogenase 2*				
	QTL	*Major quantitative trait loci*				
	Hd1	*Heading date 1*				
	LPA1	*Loose plant architecture 1*				
	MPK1, MPK2, MPK5, MPK6	*Mitogen-activated protein kinase* genes	Rice ubiquitin promoter	Rice U3 promoter	pRGEB32 binary vector	Minkenberg et al., 2017
	OsEPSPS	*5-Enolpyruvylshikimate 3-Phosphate synthase*	ZmUbi promoter	*Oryza sativa* U6 (OsU6) promoter	pCambia binary vector	Wang et al., 2017
	OsBEL	*Bentazon sensitive lethal*				
	OsPDS	*Phytoene desaturase*				
	*SWEET* genes	*Sugar efflux transporter genes* related with disease susceptibility	Maize ubiquitin 1 promoter	Rice small nuclear RNA U6	pCAMBIA1300-based destination vector pUbi-Cas9	Zhou et al., 2014
	*OsCPS4, CYP99A2*	For production of labdane-related diterpenoids, a group of phytoalexins				
	*OsKO1, OsKOL5*	Diterpenoid synthetic genes				
	*CYP76M5, CYP76M6*	*Cytochrome P450 gene*				
	*OsPDS*	Mutation resulted in albino phenotype	CaMV 35S promoter	OsU3 promoter	pCAMBIA1300	Zhang et al., 2014
	*OsPMS3*	Non-coding RNA				
	*OsDERF1*	*AP2 domain containing protein* for drought-resistant				
	*OsMSH1*	DNA mismatch repair protein; pleiotropic phenotype				
	Rice *MAPKs* (*MPK1/2/5/6*)	*Mitogen-activated protein kinase* (*MAPK*) involved in biotic and abiotic signaling pathways	Rice ubiquitin promoter plus the complete 5′ untranslated region (UBIp)	Rice U3 snoRNA promoter (U3p)	pRGEB32	Xie et al., 2015
	*FT-like* (*FTL*) genes	Premature leaf senescence	Maize ubiquitin promoter (P_ubi_) or the cauliflower mosaic virus 35S promoter (P_35S_)	U3 and U6 small nuclear RNA promoters from rice: OsU3, OsU6b, OsU6c, OsU6a	pYLCRISPR/Cas9 binary vectors based on the pCAMBIA1300 backbone	Ma et al., 2015
	*OsGSTU, OsMRP15, OsAnP*	Genes for anthocyanin accumulation				
	*OsWaxy*	Decrease amylose content				
*Triticum aestivum* (wheat)	*PDS*	*Phytoene desaturase*; albino leaf phenotype	CaMV 35S promoter	CaMV 35S promoter	pBI121	Upadhyay et al., 2013
	*INOX*	*Inositol oxygenase* enzyme				
	*TaGW2*	*Grain weight*	Ubiquitin gene promoters	Wheat U3 and U6 promoters	pBUN421-GLM	Wang et al., 2016
	*TaLpx-1*	lipoxygenase 1 related with disease resistance				
	*TaMLO*	*Mildew Resistance Locus*				
Maize (*Zea mays*)	*ZmAgo18a, ZmAgo18b*	*Argonaute 18*	Maize ubiquitin 1 gene promoter	Rice U6 small nuclear RNA gene promoters	pMCG1005 binary vector	Char et al., 2017
	*a1, a4*	*Dihydroflavonol 4-reductase or anthocyaninless*				
	*LIG*	*Liguleless1*	Ubiquitin promoter	Maize U6 polymerase III promoter	pSB11-Ubi-Cas9; U6:sgRNA	Svitashev et al., 2015
	*Ms26, Ms45*	Male fertility genes				
	*RPL, PPR*	For plant development	Maize U6 promoter	Maize U6 promoter	pCAMBIA3301	Qi et al., 2016
	lncRNAs	Two reverse overlapping long non-coding RNAs				

## Countering Off-Targets In Date Palm Genome Editing

The robustness of CRISPR/Cas9 has become unprecedented though, yet off targets are major limiting factor in the applications of this system. However, in larger eukaryotic genomes avoiding off-targets effects by choosing minimum sequences of sgRNA albeit with least similarity to unrelated sequences is nearly impossible. To dealt such problems in date palm GE can be challenging and some of the strategies have been proposed in this section. Several studies have revealed that Cas9 has the ability to cleave the target region even if several mismatches are present between sgRNA and the target sequence, thus capable to perform off-target activity. Unlike previous studies, which showed that 20 bp long sgRNA endow high level of specificity (Fu et al., 2013; Hsu et al., 2013; Pattanayak et al., 2013), new findings showed improved GE specificity by using less than 20 bp sgRNA (Fu et al., 2014). It has been empirically proven that generally up to three nucleotide mismatches, especially at 5′ end, between sgRNA and target sequence can be tolerated by CRISPR/Cas9 system (Ran et al., 2013). However, the crucial role in determining specificity is being imparted by sgRNA seed sequence—a region of 12 nucleotides following the PAM—and two nucleotides of the PAM sequence (Larson et al., 2013). Off-targets can also be substantially minimized by adding few guanidine residues at 5′ end (Cho et al., 2014) and/or by reducing the length of the sgRNA up to 17 nucleotides (Fu et al., 2014). Such reduction in size may render the RNA–DNA complex more sensitive to mismatches, perhaps by reducing binding energy at the sgRNA–DNA interface.

Another substantial way of eliminating off-targets is the use of modified Cas9 nuclease. The use of different mutant variants of Cas9 (SpCas9 VQR and VRER) (Kleinstiver et al., 2015) or Cas9 orthologs StlCas9 (derived from *Streptococcus thermophilus*) could also be used for highly efficient GE with modified PAM recognition specificity (Kleinstiver et al., 2015). A promising study showed no detectable off-targets effects in rabbits accompanying with up to 50-fold improved specificity, as compared to conventional Cas9, where single strand of target sequences was nicked by inactivating one of the two conserved nuclease domains of Cas9 (Honda et al., 2015). Such single-stranded nicks are not capable of causing mutation because of base excision repair mechanism. Cleaving the target DNA sequence by pairing the two nickases can generate double stranded sticky ends break (Mali et al., 2013; Ran et al., 2013). The obvious reason in enhanced specificity is expansion of target sequences from 20 to 40 bp by the use of two sgRNAs. However, in *Arabidopsis* and rice plants, the use of paired Cas9 nickases failed to outperform the nuclease in promoting NHEJ or HDR repair, but may reduce off-targets effects (Fauser et al., 2014; Mikami et al., 2016). Over the time, the Cas9 specificity has been improved by adopting certain strategies. In a recent study, a structure-guided approach was employed to modify alanine groove (with positively charged amino acid) that potentially disrupted the interaction of Cas9 with non-target DNA strand thus, leading to increase specificity by enhancing Cas9 reliance on the sgRNA base pairing to the target sequence (Slaymaker et al., 2016). Such Cas9 mutants (Cas9s) demonstrated enhanced, but variable, editing efficiency across multiple genomic targets with significant reduction in the off-targets (Crosetto et al., 2013). As an alternate approach, the Cas9 structure was explored to identify those amino acids, which are essential in stabilizing the target DNA strand and the 5′ end of the sgRNA (Kleinstiver et al., 2016). By mutating such amino acids, a modified Cas9 (SpCas9-HF1) was designed that not only efficiently cleave target sequence but also showed higher sensitivity to mismatches between the sgRNA and target sequences. In order to obtain desired changes free of off-targets through CRISPR/Cas9, opting such techniques could be a valuable tool in date palm GE.

## The Limitations Of Date Palm Genome Editing

Besides all the fascination, GE in date palm can have certain bottle-necks. The genomes of outcrossing species have high heterozygosity and thus, occurrence of SNPs is quite frequent and can affect the efficiency of GE in these species. Outcrossing phenomena brings high allelic heterozygosity, polymorphism, and genetic instability in the date palm genome (Jubrael et al., 2005). The genome draft of date palm cultivars Agwa, Fahal, Khalas, and Sukary has identified a SNP range of 3.85–6.63/kb in the nuclear genome (Al-Mssallem et al., 2013). Moreover, in the cultivar Khalas about 0.9 million new SNPs have also been reported. A high frequency of SNPs in the coding region is also reported from other woody perennial trees such as *Populus trichocarpa, Populus tremula*, and *Eucalyptus* (Tsai and Xue, 2015). Because, most of the plant models used for GE through CRISPR/Cas9 are highly homozygous, therefore few studies are available, which addressed sequence polymorphisms. However, the occurrence of allelic heterozygosity and sequence polymorphism may affect the GE in outcrossing, woody perennial plants (Fan et al., 2015; Tsai and Xue, 2015). The GE of *4-coumarate:CoA ligase* (4CL) genes was accomplished by successfully targeting 4CL1, 4CL2 genes with 100% biallelic mutation in transgenic popular plants. Despite of 89% sequence identity with 4CL1, the third target gene of 4CL family (4CL5) could not be mutated due to the presence of SNP near PAM sequence (Zhou et al., 2015). Different web-based programs for sgRNA design in plants have limited utility to harness outcrossing plant species. This may be due to lack of multiploidy coverage of plant genomes on the available resources. The identification of SNPs requires the genome sequence information of the respective cultivar to be used for GE. However, the cultivar chosen for whole genome sequencing may have unique biological features different from other cultivars. Thus, it is easy to speculate that the whole genome of one date palm cultivar may or may not explicitly represent the exact features of similar genes between two cultivars. The whole genome sequencing of different date palm cultivars revealed the existence of intra- and inter-varietal SNPs not only in the intergenic region but also in the coding regions (Yang et al., 2010; Khan et al., 2012; Al-Mssallem et al., 2013; Sabir et al., 2014). Under such circumstances, it is highly tricky to choose and design sgRNAs for date palm GE and other related applications using CRISPR/Cas9.

Improving the plant traits through conventional breeding is time consuming phenomena as it relies on extensive back-crossing and introgression of naturally existing genetic variation. The mounting scenario of increasing population could not be paced with conventional breeding. Nonetheless, plant breeding can be accelerated through modern GE tools especially in date palm with long breeding cycles and vegetative propagation. CRISPR/Cas9 system can be executed to eliminate genes that negatively regulate date palm quality traits and stacking molecular traits at desired locus. In gene stacking, genes can be introduced in close proximity to a desired locus into a crop with a low risk of segregation. However, such executions are tedious to achieve through classical breeding (Ainley et al., 2013). Thus, establishing the generic recipient lines through CRISPR/Cas9-based approaches would accelerate the date palm breeding program by reducing the time span.

Delivering CRISPR/Cas9 components into the date palm genome via genetic transformation of somatic embryos may possibly introduce random integration of bacterial plasmid sequences and thus, may arise current GMO interceptions. One possibility to override any transgene is a continuous back-crossing for several generations, which is not workable in date palm. It requires around 30 years accomplishing three backcrosses in date palm. The application of next-generation DNA-free CRISPR/Cas9 approaches [such as CRISPR/Cas9 ribonucleoproteins (RNPs)] may offer a plausible solution to address current GMO-regulations in such plants (Kanchiswamy, 2016; Lu et al., 2017). The CRISPR/Cas9 RNPs approach has successfully been used for grapevine and apple protoplast transformation to cope with *Erysiphe necator* and *Erwinia amylovora*, respectively by delivering CRISPR/Cas9 RNPs directly into the protoplast (Malnoy et al., 2016). The date palm protoplasts transfection may constitute dynamic and versatile CRISPR/Cas9 GE to tackle long procedure of functional analysis for the concerned traits. This method has already been deployed in human, animals, and some plants in terms of high efficiency, reduced off-targets and rapid GE (Liu et al., 2015; Woo et al., 2015; Kanchiswamy, 2016).

## Crispr-Based Future Applications In Date Palm

**FIGURE 4:**
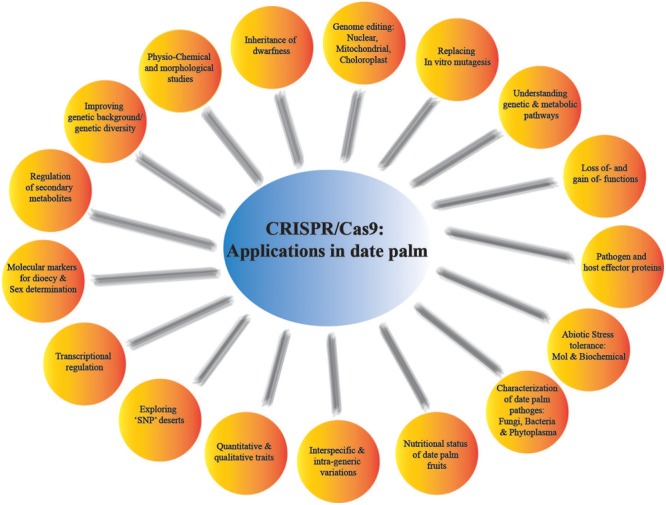
Depicted future applications of CRISPR/Cas9 in date palm genome editing and beyond.

The contemporary CRISPR versions are revolutionizing the functional genomics and molecular biology especially in characterization of the genes functions through gain and/or loss-of functions. In date palm, random mutation induced through irradiation and magnetic field has been employed to create genetic variability and selection against abiotic stresses and bayoud disease (Jain et al., 2011). However, such randomly induced mutagenesis has a severe drawback of enormous background mutation load (Braatz et al., 2017). Instead of using conventional mutagenic systems, the use of CRISPR/Cas9 system in date palm GE will definitely open up new horizons in date palm biological research. The classical mutagenic approaches are not necessarily suitable for inactivation of every gene under study due to variable nature of gene integration, which could end up in appearance of transgenes (Khatodia et al., 2016). However, site-specific mutagenesis through CRISPR/Cas9 system will enable the scientists to study gene expression of such inaccessible genes and targeting multiple loci in large date palm genome. It can also be employed to target various pathogen effectors, explore secondary metabolites in date palm fruit and improving quantitative and qualitative traits (**Figure 4**).

Moreover, sex determination in the new borne date palm progeny is not possible until the plants are matured. There are several marker-assisted approaches available to determine dioecious status of the date palm sprouts, but the exact sex determinants can be explored by CRISPR/Cas9-based approaches. By employing the gene knocking ability, CRISPR system can be used to identify genetic markers for sex determination in date palm. The targeted mutation of many negatively regulated resistance genes have been knockout or knockdown for example, OsERF922 against rice blast (Liu et al., 2012; Wang et al., 2016) and TMS5 for temperature sensitivity (Zhou et al., 2014), in rice. CRISPR/Cas9-based GE approaches provide a throughput tool for functional genomics and genetic improvement in crop plants. It can help in promoting yield components, overall plant architecture, nutrient uptake efficiency, and promoting natural plant adaptation to various stresses. Additionally, CRISPR/Cas9 system can be explored to curtail phytoplasma diseases in date palm. The additional applications may help in regulating secondary metabolites production from date palm fruits and production of biofuel from the date palm wastes (**Figure 4**).

Many agronomically important loci are located in the “SNP deserts” in the plant genome (Xu et al., 2012). SNP deserts in date palm genome recruit high density of abiotic and biotic resistance genes as compared to the whole genome (Al-Mssallem et al., 2013). These SNP deserts can be targeted to devise resistance strategies against major abiotic and biotic stresses in date palm through CRISPR/Cas9 system. Many such characters such as up-regulation of antioxidants, enhanced flavor, pests and disease resistance can be promoted through GE in date palm.

## Concluding Remarks

In future, the genetic improvement in date palm would accelerate progress against abiotic and biotic challenges for sustainable production using GE technologies. The drawbacks of conventional breeding in date palm could be circumvented by genetic manipulation and transcriptional control of various physiological and metabolic processes. The site-specific gene insertion, specificity in targeted mutation and controlled genetic manipulations can make CRISPR/Cas9 a novel tool in the date palm breeder’s kit. The availability of the date palm full genome sequence opened up new territories for yield improvement through GE and genetic manipulation as well as the applications of high-throughput genome sequencing may revolutionize date palm biotechnology by introducing targeted genetic modifications and new functionalities. It will also pave the way to improve flowering, sex determination, juvenility control and manipulation of fruit ripening, fruit quality and nutritional value in date palm. The futuristic outcomes of the application of CRISPR/Cas9 in date palm GE will not only address the basic biological questions but will definitely reduce the concerns of common people due to its non-GMO nature.

## Author Contributions

MS conceived the idea, produced the first draft of the manuscript, and helped to draw the figures. ZI collected the data, helped to draw the figures, and participated to produce the first draft of the manuscript. MT helped to formulate all the tables. MS participated to retrieve the data and helped to prepare first draft of the manuscript. MK helped to write first draft of the manuscript. AA-K and SA-K helped to finalize the final draft of the manuscript.

## Conflict of Interest Statement

The authors declare that the research was conducted in the absence of any commercial or financial relationships that could be construed as a potential conflict of interest.

## References

[B1] Ainley W. M. Sastry-Dent L. Welter M. E. Murray M. G. Zeitler B. Amora R. (2013). Trait stacking via targeted genome editing. *Plant Biotechnol. J.* 11 1126–1134. 10.1111/pbi.12107 23953646

[B2] Al-Dous E. K. George B. Al-Mahmoud M. E. Al-Jaber M. Y. Wang H. Salameh Y. M. (2011). De novo genome sequencing and comparative genomics of date palm (*Phoenix dactylifera*). *Nat. Biotechnol.* 29 521–527. 10.1038/nbt.1860 21623354

[B3] Ali Z. Abul-faraj A. Li L. Ghosh N. Piatek M. Mahjoub A. (2015). Efficient virus-mediated genome editing in plants using the CRISPR/Cas9 system. *Mol. Plant* 8 1288–1291. 10.1016/j.molp.2015.02.011 25749112

[B4] Al-Khayri J. M. Jain S. M. Johnson D. V. (2015). *Date Palm Genetic Resourse and Utilization.* Berlin: Springer.

[B5] Al-Mssallem I. S. Hu S. Zhang X. Lin Q. Liu W. Tan J. (2013). Genome sequence of the date palm *Phoenix dactylifera* L. *Nat. Commun.* 4 2274 10.1038/ncomms3274 PMC3741641 23917264

[B6] Al-Sadi A. M. Al-Jabri A. H. Al-Mazroui S. S. Al-Mahmooli I. H. (2012). Characterization and pathogenicity of fungi and oomycetes associated with root diseases of date palms in Oman. *Crop Prot.* 37 1–6. 10.1016/j.cropro.2012.02.011

[B7] Arzani A. Ashraf M. (2016). Smart engineering of genetic resources for enhanced salinity tolerance in crop plants. *Crit. Rev. Plant Sci.* 35 146–189. 10.1080/07352689.2016.1245056

[B8] Bekheet S. A. Hanafy M. S. (2011). “Date palm biotechnology,” in *Towards Sex Determination of Date Palm* eds Jain S. M. Al-Khayri J. M. Johnson D. V. (Dordrecht: Springer) 551–566. 10.1007/978-94-007-1318-5_26

[B9] Belhaj K. Chaparro-Garcia A. Kamoun S. Nekrasov V. (2013). Plant genome editing made easy: targeted mutagenesis in model and crop plants using the CRISPR/Cas system. *Plant Methods* 9 39–47. 10.1186/1746-4811-9-39 24112467 PMC3852272

[B10] Bortesi L. Fischer R. (2015). The CRISPR/Cas9 system for plant genome editing and beyond. *Biotechnol. Adv.* 33 41–52. 10.1016/j.biotechadv.2014.12.006 25536441

[B11] Bourgis F. Kilaru A. Cao X. Ngando-Ebongue G. F. Drira N. Ohlrogge J. B. (2011). Comparative transcriptome and metabolite analysis of oil palm and date palm mesocarp that differ dramatically in carbon partitioning. *Proc. Natl. Acad. Sci. U.S.A.* 108 12527–12532. 10.1073/pnas.1106502108 21709233 PMC3145713

[B12] Braatz J. Harloff H.-J. Mascher M. Stein N. Himmelbach A. Jung C. (2017). CRISPR-Cas9 targeted mutagenesis leads to simultaneous modification of different homoeologous gene copies in polyploid oilseed rape (*Brassica napus*). *Plant Physiol.* 174 935–942. 10.1104/pp.17.00426 28584067 PMC5462057

[B13] Brooks C. Nekrasov V. Lippman Z. B. Van Eck J. (2014). Efficient gene editing in tomato in the first generation using the clustered regularly interspaced short palindromic repeats/CRISPR-associated 9 System. *Plant Physiol.* 166 1292–1297. 10.1104/pp.114.247577 25225186 PMC4226363

[B14] Butler N. M. Baltes N. J. Voytas D. F. Douches D. S. (2016). Geminivirus-mediated genome editing in potato (*Solanum tuberosum* L.) using sequence-specific nucleases. *Front. Plant Sci.* 7:1045 10.3389/fpls.2016.01045 PMC4955380 27493650

[B15] Char S. N. Neelakandan A. K. Nahampun H. Frame B. Main M. Spalding M. H. (2017). An Agrobacterium-delivered CRISPR/Cas9 system for high-frequency targeted mutagenesis in maize. *Plant Biotechnol. J.* 15 257–268. 10.1111/pbi.12611 27510362 PMC5259581

[B16] Cho S. W. Kim S. Kim Y. Kweon J. Kim H. S. Bae S. (2014). Analysis of off-target effects of CRISPR/Cas-derived RNA-guided endonucleases and nickases. *Genome Res.* 24 132–141. 10.1101/gr.162339.113 24253446 PMC3875854

[B17] Christian M. Qi Y. Zhang Y. Voytas D. F. (2013). Targeted mutagenesis of *Arabidopsis thaliana* using engineered TAL effector nucleases. *G3 (Bethesda)* 3 1697–1705. 10.1534/g3.113.007104 23979944 PMC3789794

[B18] Crosetto N. Mitra A. Silva M. J. Bienko M. Dojer N. Wang Q. (2013). Nucleotide-resolution DNA double-strand break mapping by next-generation sequencing. *Nat. Methods* 10 361–365. 10.1038/nmeth.2408 23503052 PMC3651036

[B19] Curtin S. J. Anderson J. E. Starker C. G. Baltes N. J. Mani D. Voytas D. F. (2013). “Targeted mutagenesis for functional analysis of gene duplication in legumes,” in *Legume Genomics: Methods and Protocols* ed. Rose R. J. (Totowa, NJ: Humana Press) 25–42. 10.1007/978-1-62703-613-9_3 23996306

[B20] Dahlem T. J. Hoshijima K. Jurynec M. J. Gunther D. Starker C. G. Locke A. S. (2012). Simple methods for generating and detecting locus-specific mutations induced with TALENs in the zebrafish genome. *PLoS Genet.* 8:e1002861 10.1371/journal.pgen.1002861 PMC3420959 22916025

[B21] Ding Y. Li H. Chen L. L. Xie K. (2016). Recent advances in genome editing using CRISPR/Cas9. *Front. Plant Sci.* 7:703 10.3389/fpls.2016.00703 PMC4877526 27252719

[B22] Dong F. Xie K. Chen Y. Yang Y. Mao Y. (2017). Polycistronic tRNA and CRISPR guide-RNA enables highly efficient multiplexed genome engineering in human cells. *Biochem. Biophys. Res. Commun.* 482 889–895. 10.1016/j.bbrc.2016.11.129 27890617 PMC5284736

[B23] El Rabey H. A. Al-Malki A. L. Abulnaja K. O. Rohde W. (2015). Proteome analysis for understanding abiotic stress (salinity and drought) tolerance in date palm (*Phoenix dactylifera* L.). *Int. J. Genomics* 2015 1–11. 10.1155/2015/407165 PMC4488584 26167472

[B24] Fan D. Liu T. Li C. Jiao B. Li S. Hou Y. (2015). Efficient CRISPR/Cas9-mediated targeted mutagenesis in populus in the first generation. *Sci. Rep.* 5:12217 10.1038/srep12217 PMC4507398 26193631

[B25] Fang Y. Tyler B. M. (2016). Efficient disruption and replacement of an effector gene in the oomycete *Phytophthora sojae* using CRISPR/Cas9. *Mol. Plant Pathol.* 17 127–139. 10.1111/mpp.12318 26507366 PMC6638440

[B26] Fang Y. Wu H. Zhang T. Yang M. Yin Y. Pan L. (2012). A complete sequence and transcriptomic analyses of date palm (*Phoenix dactylifera* L.) mitochondrial genome. *PLoS ONE* 7:e37164 10.1371/journal.pone.0037164 PMC3360038 22655034

[B27] Fauser F. Schiml S. Puchta H. (2014). Both CRISPR/Cas-based nucleases and nickases can be used efficiently for genome engineering in *Arabidopsis thaliana*. *Plant J.* 79 348–359. 10.1111/tpj.12554 24836556

[B28] Feng Z. Mao Y. Xu N. Zhang B. Wei P. Yang D. L. (2014). Multigeneration analysis reveals the inheritance, specificity, and patterns of CRISPR/Cas-induced gene modifications in *Arabidopsis*. *Proc. Natl. Acad. Sci. U.S.A.* 111 4632–4637. 10.1073/pnas.1400822111 24550464 PMC3970504

[B29] Ferry M. Gomez S. (2002). The red palm weevil in the Mediterranean area. *J. Int. Palm Soc.* 46 172–178.

[B30] Fu Y. Foden J. A. Khayter C. Maeder M. L. Reyon D. Joung J. K. (2013). High frequency off-target mutagenesis induced by CRISPR-Cas nucleases in human cells. *Nat. Biotechnol.* 31 822–826. 10.1038/nbt.2623 23792628 PMC3773023

[B31] Fu Y. Sander J. D. Reyon D. Cascio V. M. Joung J. K. (2014). Improving CRISPR-Cas nuclease specificity using truncated guide RNAs. *Nat. Biotechnol.* 32 279–284. 10.1038/nbt.2808 24463574 PMC3988262

[B32] Gao J. Wang G. Ma S. Xie X. Wu X. Zhang X. (2015). CRISPR/Cas9-mediated targeted mutagenesis in *Nicotiana tabacum*. *Plant Mol. Biol.* 87 99–110. 10.1007/s11103-014-0263-0 25344637

[B33] Gilbert L. A. Larson M. H. Morsut L. Liu Z. Brar G. A. Torres S. E. (2013). CRISPR-mediated modular RNA-guided regulation of transcription in eukaryotes. *Cell* 154 442–451. 10.1016/j.cell.2013.06.044 23849981 PMC3770145

[B34] Hazzouri K. M. Flowers J. M. Visser H. J. Khierallah H. S. M. Rosas U. Pham G. M. (2015). Whole genome re-sequencing of date palms yields insights into diversification of a fruit tree crop. *Nat. Commun.* 6 8824 10.1038/ncomms9824 PMC4667612 26549859

[B35] Honda A. Hirose M. Sankai T. Yasmin L. Yuzawa K. Honsho K. (2015). Single-step generation of rabbits carrying a targeted allele of the tyrosinase gene using CRISPR/Cas9. *Exp. Anim.* 64 31–37. 10.1538/expanim.14-0034 25195632 PMC4329513

[B36] Hsu P. D. Scott D. A. Weinstein J. A. Ran F. A. Konermann S. Agarwala V. (2013). DNA targeting specificity of RNA-guided Cas9 nucleases. *Nat. Biotechnol.* 31 827–832. 10.1038/nbt.2647 23873081 PMC3969858

[B37] Hua Y. Wang C. Huang J. Wang K. (2017). A simple and efficient method for CRISPR/Cas9-induced mutant screening. *J. Genet. Gen.* 44 207–213. 10.1016/j.jgg.2017.03.005 28416245

[B38] Iqbal Z. Sattar M. N. Shafiq M. (2016). CRISPR/Cas9: a tool to circumscribe cotton leaf curl disease. *Front. Plant Sci* 7:475 10.3389/fpls.2016.00475 PMC4828465 27148303

[B39] Jacobs T. B. LaFayette P. R. Schmitz R. J. Parrott W. A. (2015). Targeted genome modifications in soybean with CRISPR/Cas9. *BMC Biotechnol.* 15:16 10.1186/s12896-015-0131-2 PMC4365529 25879861

[B40] Jain S. M. Al-Khayri J. Johnson D. V. (2011). *Date Palm Biotechnology.* New York, NY: Springer Science+Business Media B.V. 10.1007/978-94-007-1318-5

[B41] Jia H. Orbovic V. Jones J. B. Wang N. (2016). Modification of the PthA4 effector binding elements in Type I CsLOB1 promoter using Cas9/sgRNA to produce transgenic Duncan grapefruit alleviating XccΔpthA4:dCsLOB1.3 *infection*. *Plant Biotechnol. J.* 14 1291–1301. 10.1111/pbi.12495 27071672 PMC11389130

[B42] Jia H. Wang N. (2014). Targeted genome editing of sweet orange using Cas9/sgRNA. *PLoS ONE* 9:e93806 10.1371/journal.pone.0093806 PMC3977896 24710347

[B43] Jiang W. Zhou H. Bi H. Fromm M. Yang B. Weeks D. P. (2013). Demonstration of CRISPR/Cas9/sgRNA-mediated targeted gene modification in Arabidopsis, tobacco, sorghum and rice. *Nucleic Acids Res.* 41:e188 10.1093/nar/gkt780 PMC3814374 23999092

[B44] Jubrael J. M. S. Udupa S. M. Baum M. (2005). Assessment of AFLP-based genetic relationships among date palm (*Phoenix dactylifera* L.) varieties of Iraq. *J. Am. Soc. Hortic. Sci.* 130 442–447.

[B45] Kanchiswamy C. N. (2016). DNA-free genome editing methods for targeted crop improvement. *Plant Cell Rep.* 35 1469–1474. 10.1007/s00299-016-1982-2 27100964

[B46] Kanda Y. Yokotani N. Maeda S. Nishizawa Y. Kamakura T. Mori M. (2017). The receptor-like cytoplasmic kinase BSR1 mediates chitin-induced defense signaling in rice cells. *Biosci. Biotechnol. Biochem.* 81 1497–1502. 10.1080/09168451.2017.1325710 28521637

[B47] Khan A. Khan I. A. Heinze B. Azim M. K. (2012). The chloroplast genome sequence of date palm (*Phoenix dactylifera* L. cv. ‘Aseel’). *Plant Mol. Biol. Rep.* 30 666–678. 10.1007/s11105-011-0373-7

[B48] Khan M. S. Khalid A. M. Malik K. A. (2005). Phage phiC31 integrase: a new tool in plastid genome engineering. *Trends Plant Sci.* 10 1–3. 10.1016/j.tplants.2004.11.001 15642516

[B49] Khatodia S. Bhatotia K. Passricha N. Khurana S. M. P. Tuteja N. (2016). The CRISPR/Cas genome-editing tool: application in improvement of crops. *Front. Plant Sci.* 7:506 10.3389/fpls.2016.00506 PMC4835450 27148329

[B50] Khatodia S. Bhatotia K. Tuteja N. (2017). Development of CRISPR/Cas9 mediated virus resistance in agriculturally important crops. *Bioengineered* 8 274–279. 10.1080/21655979.2017.1297347 28581909 PMC5470520

[B51] Kleinstiver B. P. Pattanayak V. Prew M. S. Tsai S. Q. Nguyen N. T. Zheng Z. (2016). High-fidelity CRISPR–Cas9 nucleases with no detectable genome-wide off-target effects. *Nature* 529 490–495. 10.1038/nature16526 26735016 PMC4851738

[B52] Kleinstiver B. P. Prew M. S. Tsai S. Q. Topkar V. V. Nguyen N. T. Zheng Z. (2015). Engineered CRISPR-Cas9 nucleases with altered PAM specificities. *Nature* 523 481–485. 10.1038/nature14592 26098369 PMC4540238

[B53] Larson M. H. Gilbert L. A. Wang X. Lim W. A. Weissman J. S. Qi L. S. (2013). CRISPR interference (CRISPRi) for sequence-specific control of gene expression. *Nat. Protoc.* 8 2180–2196. 10.1038/nprot.2013.132 24136345 PMC3922765

[B54] Lee C. M. Cradick T. J. Fine E. J. Bao G. (2016). Nuclease target site selection for maximizing on-target activity and minimizing off-target effects in genome editing. *Mol. Ther.* 24 475–487. 10.1038/mt.2016.1 26750397 PMC4786925

[B55] Lee D. W. Kim J. K. Lee S. Choi S. Kim S. Hwang I. (2008). *Arabidopsis* nuclear-encoded plastid transit peptides contain multiple sequence subgroups with distinctive chloroplast-targeting sequence motifs. *Plant Cell* 20 1603–1622. 10.1105/tpc.108.060541 18552198 PMC2483360

[B56] Lei S. Q. Larson Matthew H. Gilbert Luke A. Doudna Jennifer A. (2013). Repurposing CRISPR as an RNA-guided platform for sequence-specific control of gene expression. *Cell* 152 1173–1183. 10.1016/j.cell.2013.02.022 23452860 PMC3664290

[B57] Li J. F. Aach J. Norville J. E. McCormack M. Zhang D. Bush J. (2013). Multiplex and homologous recombination-mediated plant genome editing via guide RNA/Cas9. *Nat. Biotechnol.* 31 688–691. 10.1038/nbt.2654 23929339 PMC4078740

[B58] Li R. Li R. Li X. Fu D. Zhu B. Tian H. (2017). Multiplexed CRISPR/Cas9-mediated metabolic engineering of γ-aminobutyric acid levels in *Solanum lycopersicum*. *Plant Biotechnol. J.* 10.1111/pbi.12781 [Epub ahead of print]. PMC5787826 28640983

[B59] Liu D. Chen X. Liu J. Ye J. Guo Z. (2012). The rice ERF transcription factor OsERF922 negatively regulates resistance to *Magnaporthe oryzae* and salt tolerance. *J. Exp. Bot.* 63 3899–3911. 10.1093/jxb/ers079 22442415 PMC3388842

[B60] Liu J. Gaj T. Yang Y. Wang N. Shui S. Kim S. (2015). Efficient delivery of nuclease proteins for genome editing in human stem cells and primary cells. *Nat. Protoc.* 10 1842–1859. 10.1038/nprot.2015.117 26492140

[B61] Lowder L. G. Paul J. W. Qi Y. (2017). “Multiplexed transcriptional activation or repression in plants using CRISPR-dCas9-based systems,” in *Plant Gene Regulatory Networks: Methods and Protocols* eds Kaufmann K. Mueller-Roeber B. (New York, NY: Springer) 167–184. 10.1007/978-1-4939-7125-1_12 28623586

[B62] Lu H. P. Liu S. M. Xu S. L. Chen W. Y. Zhou X. Tan Y. Y. (2017). CRISPR-S: an active interference element for a rapid and inexpensive selection of genome-edited, transgene-free rice plants. *Plant Biotechnol. J.* 10.1111/pbi.12788 [Epub ahead of print]. PMC5633759 28688132

[B63] Ma X. Zhang Q. Zhu Q. Liu W. Chen Y. Qiu R. (2015). A robust CRISPR/Cas9 system for convenient, high-efficiency multiplex genome editing in monocot and dicot plants. *Mol. Plant* 8 1274–1284. 10.1016/j.molp.2015.04.007 25917172

[B64] Ma X. Zhu Q. Chen Y. Liu Y. G. (2016). CRISPR/Cas9 platforms for genome editing in plants: developments and applications. *Mol. Plant* 9 961–974. 10.1016/j.molp.2016.04.009 27108381

[B65] Mahmoudi H. Hosseininia G. Azadi H. Fatemi M. (2008). Enhancing date palm processing, marketing and pest control through organic culture. *J. Organ. Syst.* 3 29–39.

[B66] Mali P. Aach J. Stranges P. B. Esvelt K. M. Moosburner M. Kosuri S. (2013). Cas9 transcriptional activators for target specificity screening and paired nickases for cooperative genome engineering. *Nat. Biotechnol.* 31 833–838. 10.1038/nbt.2675 23907171 PMC3818127

[B67] Malnoy M. Viola R. Jung M. H. Koo O. J. Kim S. Kim J. S. (2016). DNA-free genetically edited grapevine and apple protoplast using CRISPR/Cas9 ribonucleoproteins. *Front. Plant Sci.* 7:1904 10.3389/fpls.2016.01904 PMC5170842 28066464

[B68] Mao Y. Zhang H. Xu N. Zhang B. Gao F. Zhu J. K. (2013). Application of the CRISPR-Cas system for efficient genome engineering in plants. *Mol. Plant* 6 2008–2011. 10.1093/mp/sst121 23963532 PMC3916745

[B69] Mathew L. S. Seidel M. A. George B. Mathew S. Spannagl M. Haberer G. (2015). A genome-wide survey of date palm cultivars supports two major subpopulations in *Phoenix dactylifera*. *G3 (Bethesda)* 5 1429–1438. 10.1534/g3.115.018341 25957276 PMC4502377

[B70] Mercx S. Smargiasso N. Chaumont F. De Pauw E. Boutry M. Navarre C. (2017). Inactivation of the β(1,2)-xylosyltransferase and the α(1,3)-fucosyltransferase genes in *Nicotiana tabacum* BY-2 cells by a multiplex CRISPR/Cas9 strategy results in glycoproteins without plant-specific glycans. *Front. Plant Sci.* 8:403 10.3389/fpls.2017.00403 PMC5366340 28396675

[B71] Miao J. Guo D. Zhang J. Huang Q. Qin G. Zhang X. (2013). Targeted mutagenesis in rice using CRISPR-Cas system. *Cell Res.* 23 1233–1236. 10.1038/cr.2013.123 23999856 PMC3790239

[B72] Mikami M. Toki S. Endo M. (2016). Precision targeted mutagenesis via Cas9 paired nickases in rice. *Plant Cell Physiol.* 57 1058–1068. 10.1093/pcp/pcw049 26936792 PMC4867050

[B73] Minkenberg B. Xie K. Yang Y. (2017). Discovery of rice essential genes by characterizing a CRISPR-edited mutation of closely related rice MAP kinase genes. *Plant J.* 89 636–648. 10.1111/tpj.13399 27747971

[B74] Mokhtar M. M. Adawy S. S. El-Assal S. E.-D. S. Hussein E. H. A. (2016). Genic and intergenic SSR database generation, SNPs determination and pathway annotations, in date palm (*Phoenix dactylifera* L.). *PLoS ONE* 11:e0159268 10.1371/journal.pone.0159268 PMC4951042 27434138

[B75] Mousavi M. Mousavi A. Habashi A. A. Dehsara B. (2014). Genetic transformation of date palm (*Phoenix dactylifera* L. cv. ‘Estamaran’) via particle bombardment. *Mol. Biol. Rep.* 41 8185–8194. 10.1007/s11033-014-3720-6 25200434

[B76] Nekrasov V. Wang C. Win J. Lanz C. Weigel D. Kamoun S. (2017). Rapid generation of a transgene-free powdery mildew resistant tomato by genome deletion. *Sci. Rep.* 7 482 10.1038/s41598-017-00578-x PMC5428673 28352080

[B77] Nishitani C. Hirai N. Komori S. Wada M. Okada K. Osakabe K. (2016). Efficient genome editing in apple using a CRISPR/Cas9 system. *Sci. Rep.* 6 1–8. 10.1038/srep31481 27530958 PMC4987624

[B78] Pater S. Pinas J. E. Hooykaas P. J. J. Zaal B. J. (2013). ZFN-mediated gene targeting of the Arabidopsis protoporphyrinogen oxidase gene through *Agrobacterium*-mediated floral dip transformation. *Plant Biotechnol. J.* 11 510–515. 10.1111/pbi.12040 23279135 PMC3719044

[B79] Pattanayak V. Lin S. Guilinger J. P. Ma E. Doudna J. A. Liu D. R. (2013). High-throughput profiling of off-target DNA cleavage reveals RNA-programmed Cas9 nuclease specificity. *Nat. Biotechnol.* 31 839–843. 10.1038/nbt.2673 23934178 PMC3782611

[B80] Peng A. Chen S. Lei T. Xu L. He Y. Wu L. (2017). Engineering canker-resistant plants through CRISPR/Cas9-targeted editing of the susceptibility gene CsLOB1 promoter in citrus. *Plant Biotechnol. J.* 10.1111/pbi.12733 [Epub ahead of print]. PMC5698050 28371200

[B81] Peterson B. A. Haak D. C. Nishimura M. T. Teixeira P. J. P. L. James S. R. Dangl J. L. (2016). Genome-wide assessment of efficiency and specificity in CRISPR/Cas9 mediated multiple site targeting in Arabidopsis. *PLoS ONE* 11:e0162169 10.1371/journal.pone.0162169 PMC5021288 27622539

[B82] Pintaud J. C. Ludeña B. Aberlenc Bertossi F. Zehdi S. Gros Balthazard M. Ivorra S. (2011). “Biogeography of the date palm (Phoenix dactylifera L., *Arecaceae*): insights on the origin and on the structure of modern diversity,” in *Proceedings of the Ist International Symposium on Date Palm* Algeria 19–38.

[B83] Qi W. Zhu T. Tian Z. Li C. Zhang W. Song R. (2016). High-efficiency CRISPR/Cas9 multiplex gene editing using the glycine tRNA-processing system-based strategy in maize. *BMC Biotechnol.* 16:58 10.1186/s12896-016-0289-2 PMC4982333 27515683

[B84] Qi Y. Li X. Zhang Y. Starker C. G. Baltes N. J. Zhang F. (2013). Targeted deletion and inversion of tandemly arrayed genes in *Arabidopsis thaliana* using zinc finger nucleases. *G3 (Bethesda)* 3 1707–1715. 10.1534/g3.113.006270 23979943 PMC3789795

[B85] Ran F. A. Hsu Patrick D. Lin C. Y. Gootenberg Jonathan S. Konermann S. (2013). Double nicking by RNA-guided CRISPR Cas9 for enhanced genome editing specificity. *Cell* 154 1380–1389. 10.1016/j.cell.2013.08.021 23992846 PMC3856256

[B86] Rekik I. Chaabene Z. Grubb C. D. Drira N. Cheour F. Elleuch A. (2015). In silico characterization and molecular modeling of double-strand break repair protein MRE11 from *Phoenix dactylifera* vs deglet nour. *Theor. Biol. Med. Model.* 12 23 10.1186/s12976-015-0013-2 PMC4635681 26541955

[B87] Sabir J. S. M. Arasappan D. Bahieldin A. Abo-Aba S. Bafeel S. Zari T. A. (2014). Whole mitochondrial and plastid genome SNP analysis of nine date palm cultivars reveals plastid heteroplasmy and close phylogenetic relationships among cultivars. *PLoS ONE* 9:e94158 10.1371/journal.pone.0094158 PMC3981771 24718264

[B88] Schiml S. Fauser F. Puchta H. (2014). The CRISPR/Cas system can be used as nuclease for in planta gene targeting and as paired nickases for directed mutagenesis in Arabidopsis resulting in heritable progeny. *Plant J.* 80 1139–1150. 10.1111/tpj.12704 25327456

[B89] Sedra M. (2007). “Bayoud disease of date palm in North Africa: recent distribution and remarks about its characterization, diagnosis and origin,” in *Proceedings of the Fourth Symposium Date Palm* Hofuf: King Faisal University.

[B90] Shan Q. Wang Y. Li J. Zhang Y. Chen K. Liang Z. (2013). Targeted genome modification of crop plants using a CRISPR-Cas system. *Nat. Biotechnol.* 31 686–688. 10.1038/nbt.2650 23929338

[B91] Shen L. Hua Y. Fu Y. Li J. Liu Q. Jiao X. (2017). Rapid generation of genetic diversity by multiplex CRISPR/Cas9 genome editing in rice. *Sci. China Life Sci.* 60 506–515. 10.1007/s11427-017-9008-8 28349304

[B92] Shukla V. K. Doyon Y. Miller J. C. DeKelver R. C. Moehle E. A. Worden S. E. (2009). Precise genome modification in the crop species *Zea mays* using zinc-finger nucleases. *Nature* 459 437–441. 10.1038/nature07992 19404259

[B93] Siebert R. Puchta H. (2002). Efficient repair of genomic double-strand breaks by homologous recombination between directly repeated sequences in the plant genome. *Plant Cell* 14 1121–1131. 10.1105/tpc.001727 12034901 PMC150611

[B94] Slaymaker I. M. Gao L. Zetsche B. Scott D. A. Yan W. X. Zhang F. (2016). Rationally engineered Cas9 nucleases with improved specificity. *Science* 351 84–88. 10.1126/science.aad5227 26628643 PMC4714946

[B95] Stracker T. H. Petrini J. H. J. (2011). The MRE11 complex: starting from the ends. *Nat. Rev. Mol. Cell Biol.* 12 90–103. 10.1038/nrm3047 21252998 PMC3905242

[B96] Sun Z. Li N. Huang G. Xu J. Pan Y. Wang Z. (2013). Site-specific gene targeting using transcription activator-like effector (TALE)-based nuclease in *Brassica oleracea*. *J. Integrat. Plant Biol.* 55 1092–1103. 10.1111/jipb.12091 23870552

[B97] Svitashev S. Young J. K. Schwartz C. Gao H. Falco S. C. Cigan A. M. (2015). Targeted mutagenesis, precise gene editing, and site-specific gene insertion in maize using Cas9 and guide RNA. *Plant Physiol.* 169 931–945. 10.1104/pp.15.00793 26269544 PMC4587463

[B98] Townsend J. A. Wright D. A. Winfrey R. J. Fu F. Maeder M. L. Joung J. K. (2009). High-frequency modification of plant genes using engineered zinc-finger nucleases. *Nature* 459 442–445. 10.1038/nature07845 19404258 PMC2743854

[B99] Tsai C. J. Xue L. J. (2015). CRISPRing into the woods. *GM Crops Food* 6 206–215. 10.1080/21645698.2015.1091553 26357840 PMC5033219

[B100] Upadhyay S. K. Kumar J. Alok A. Tuli R. (2013). RNA-guided genome editing for target gene mutations in wheat. *G3 (Bethesda)* 3 2233–2238. 10.1534/g3.113.008847 24122057 PMC3852385

[B101] Wang F. Wang C. Liu P. Lei C. Hao W. Gao Y. (2016). Enhanced rice blast resistance by CRISPR/Cas9-targeted mutagenesis of the ERF transcription factor gene OsERF922. *PLoS ONE* 11:e0154027 10.1371/journal.pone.0154027 PMC4846023 27116122

[B102] Wang M. Mao Y. Lu Y. Tao X. Zhu J.-K. (2017). Multiplex gene editing in rice using the CRISPR-Cpf1 system. *Mol. Plant* 10 1011–1013. 10.1016/j.molp.2017.03.001 28315752

[B103] Wendt T. Holm P. B. Starker C. G. Christian M. Voytas D. F. Brinch-Pedersen H. (2013). TAL effector nucleases induce mutations at a pre-selected location in the genome of primary barley transformants. *Plant Mol. Biol.* 83 279–285. 10.1007/s11103-013-0078-4 23689819 PMC7880306

[B104] Woo J. W. Kim J. Kwon S. I. Corvalan C. Cho S. W. Kim H. (2015). DNA-free genome editing in plants with preassembled CRISPR-Cas9 ribonucleoproteins. *Nat. Biotechnol.* 33 1162–1164. 10.1038/nbt.3389 26479191

[B105] Xiao Y. Xia W. Yang Y. Mason A. S. Lei X. Ma Z. (2013). Characterization and evolution of conserved microRNA through duplication events in date palm (*Phoenix dactylifera*). *PLoS ONE* 8:e71435 10.1371/journal.pone.0071435 PMC3738527 23951162

[B106] Xie K. Minkenberg B. Yang Y. (2015). Boosting CRISPR/Cas9 multiplex editing capability with the endogenous tRNA-processing system. *Proc. Natl. Acad. Sci. U.S.A.* 112 3570–3575. 10.1073/pnas.1420294112 25733849 PMC4371917

[B107] Xie K. Yang Y. (2013). RNA-guided genome editing in plants using A CRISPR-Cas system. *Mol. Plant* 6 1975–1983. 10.1093/mp/sst119 23956122

[B108] Xin C. Liu W. Lin Q. Zhang X. Cui P. Li F. (2015). Profiling microRNA expression during multi-staged date palm (*Phoenix dactylifera* L.) fruit development. *Genomics* 105 242–251. 10.1016/j.ygeno.2015.01.004 25638647

[B109] Xu X. Liu X. Ge S. Jensen J. D. Hu F. Li X. (2012). Resequencing 50 accessions of cultivated and wild rice yields markers for identifying agronomically important genes. *Nat. Biotechnol.* 30 105–111. 10.1038/nbt.2050 22158310

[B110] Yaish M. W. Sunkar R. Zheng Y. Ji B. Al-Yahyai R. Farooq S. A. (2015). A genome-wide identification of the miRNAome in response to salinity stress in date palm (*Phoenix dactylifera* L.). *Front. Plant Sci.* 6:946 10.3389/fpls.2015.00946 PMC4633500 26594218

[B111] Yang M. Zhang X. Liu G. Yin Y. Chen K. Yun Q. (2010). The complete chloroplast genome sequence of date palm (*Phoenix dactylifera* L.). *PLoS ONE* 5:e12762 10.1371/journal.pone.0012762 PMC2939885 20856810

[B112] Yin Y. Zhang X. Fang Y. Pan L. Sun G. Xin C. (2012). High-throughput sequencing-based gene profiling on multi-staged fruit development of date palm (*Phoenix dactylifera* L.). *Plant Mol. Biol.* 78 617–626. 10.1007/s11103-012-9890-5 22351158 PMC3313043

[B113] Zhang F. Voytas D. F. (2011). “Targeted mutagenesis in *Arabidopsis* using zinc-finger nucleases,” in *Plant Chromosome Engineering: Methods and Protocols* ed. Birchler J. A. (Totowa, NJ: Humana Press) 167–177. 10.1007/978-1-61737-957-4_9 21181530

[B114] Zhang G. Pan L. Yin Y. Liu W. Huang D. Zhang T. (2012). Large-scale collection and annotation of gene models for date palm (*Phoenix dactylifera* L.). *Plant Mol. Biol.* 79 521–536. 10.1007/s11103-012-9924-z 22736259 PMC3402680

[B115] Zhang H. Zhang J. Wei P. Zhang B. Gou F. Feng Z. (2014). The CRISPR/Cas9 system produces specific and homozygous targeted gene editing in rice in one generation. *Plant Biotechnol. J.* 12 797–807. 10.1111/pbi.12200 24854982

[B116] Zhang Y. Bai Y. Wu G. Zou S. Chen Y. Gao C. (2017). Simultaneous modification of three homoeologs of TaEDR1 by genome editing enhances powdery mildew resistance in wheat. *Plant J.* 91 714–724. 10.1111/tpj.13599 28502081

[B117] Zhang Y. Liang Z. Zong Y. Wang Y. Liu J. Chen K. (2016). Efficient and transgene-free genome editing in wheat through transient expression of CRISPR/Cas9 DNA or RNA. *Nat. Commun.* 7 1–8. 10.1038/ncomms12617 PMC5007326 27558837

[B118] Zhang Y. Su J. Duan S. Ao Y. Dai J. Liu J. (2011). A highly efficient rice green tissue protoplast system for transient gene expression and studying light/chloroplast-related processes. *Plant Methods* 7 30–38. 10.1186/1746-4811-7-30 21961694 PMC3203094

[B119] Zhao Y. Williams R. Prakash C. S. He G. (2012). Identification and characterization of gene-based SSR markers in date palm (*Phoenix dactylifera* L.). *BMC Plant Biol.* 12:237 10.1186/1471-2229-12-237 PMC3568718 23241238

[B120] Zhang Y. Zhang F. Li X. Baller J. A. Qi Y. Starker C. G. (2013). Transcription activator-like effector nucleases enable efficient plant genome engineering. *Plant Physiol.* 161 20–27. 10.1104/pp.112.205179 23124327 PMC3532252

[B121] Zhang Z. Mao Y. Ha S. Liu W. Botella J. R. Zhu J. K. (2016). A multiplex CRISPR/Cas9 platform for fast and efficient editing of multiple genes in Arabidopsis. *Plant Cell Rep.* 35 1519–1533. 10.1007/s00299-015-1900-z 26661595 PMC5512712

[B122] Zhou H. Liu B. Weeks D. P. Spalding M. H. Yang B. (2014). Large chromosomal deletions and heritable small genetic changes induced by CRISPR/Cas9 in rice. *Nucleic Acids Res.* 12 10903–10914. 10.1093/nar/gku806 PMC4176183 25200087

[B123] Zhou X. Jacobs T. B. Xue L. J. Harding S. A. Tsai C. J. (2015). Exploiting SNPs for biallelic CRISPR mutations in the outcrossing woody perennial Populus reveals 4-coumarate:CoA ligase specificity and redundancy. *New Phytol.* 208 298–301. 10.1111/nph.13470 25970829

