# Supplementary material for: CRISPR/Cas9: A Practical Approach in Date Palm Genome Editing
Source: Front Plant Sci. 2017 Aug 23;8:1469. doi: 10.3389/fpls.2017.01469 (PMC5572371; doi:10.3389/fpls.2017.01469)
Supplement: Supplementary file 1 [file Table_1.docx]

Supplementary Material

CRISPR/Cas9: A practical approach in date palm genome editing

**Muhammad Naeem Sattar ^1,¶,*^, Zafar Iqbal ^2,¶^, Muhammad Nouman Tahir ^3^, Muhammad Shafiq Shahid ^4^, Muhammad Khurshid ^5^, Abdullatif A. Al-Khateeb ^6^, Suliman A. Al-Khateeb ***

*** Correspondence:** Muhammad Naeem Sattar [Naeem.sattar1177@gmail.com](mailto:Naeem.sattar1177@gmail.com)

# Supplementary Table 1: List of major plant pathogens infecting date palm in different parts of the date palm growing regions.

| **Biotic stress** | **Causal organism** | **Country** | **Reference** |
| --- | --- | --- | --- |
| Al-Wijam disease | Phytoplasma | Egypt, Kuwait, Saudi Arabia,  Sudan, USA | (Ammar et al., 2005; Alhudaib et al., 2008; Alhudaib et al., 2014; AlKhazindar, 2014; Gurr et al., 2015; Harrison & Elliott, 2016) |
| Bayoud disease | *Fusarium oxysporum* | Algeria, Mauritania, Morocco, | (Djerbi, 1998; Touzi, 2007; (Bouguedoura et al., 2008) |
| Black scorch | *Thielaviopsis paradoxa*  *Chalaropsis radiciola* | Algeria, Egypt, Iraq, Israel, Mauritania, Palestine, Qatar, Saudi Arabia, Syria,  Tunisia, UAE, Yemen | (Lahaam, 2005; Al-Sakaff, 2012; Saeed et al., 2016) |
| Brown leaf spot | *Mycosphaerella tassiana*, *Alternaria* spp. | Algeria, Egypt, Iraq, Morocco, Oman, Tunisia, UAE |  |
| Diplodia | *Diplodia phoenicum* | Egypt, Iraq, Israel, Kuwait, Libya, Morocco, Oman, Palestine, Saudi Arabia, Sudan, Tunisia, UAE, Yemen | (Lahaam, 2005; Abul-Suad, 2011; Al-Sakaff, 2012) |
| Graphiola leaf spot | *Graphiola phoenicis* | Egypt, India, Iran, Iraq, Kuwait, Libya, Mauritania, Morocco, Oman, Pakistan, Palestine, Saudi Arabia, Spain, Sudan, Tunisia, Yemen | (Al-Sakaff, 2012) |
| Khamedj or Inflorescence rot | *Mauginiella scattae* | Algeria, Bahrain, Egypt,  Iraq, Israel, Kuwait, Libya, Mauritania, Morocco, Saudi Arabia, Spain, Tunisia | (Abdullah et al., 2005; Al-badran, 2008) |
| Omphalia root rot | *Omphalia* *tralucida* | Mauritania |  |
| Belâat | *Phytophtora* sp. | Algeria, Iraq, Morocco, Tunisia, UAE |  |
| Fruit rot | *Aspergillus niger*, *Pencellium* sp., *Alternaria* sp., *Fusarium* sp. | Algeria, Palestine, UAE | (Lahaam, 2005) |
| **Insects** |  |  |  |
| Ghobar mite (Bunch fading disorder) | *Oligonychus afrasiaticus* McGregor, *O. pratensis* Banks | Algeria, Bahrain, Egypt, GCC countries, Iran, Morocco, Syria, Tunisia, Yemen | (Ba-Angood and Bass’haih (2000; Palevsky et al., 2004; Al-Sewidi & Al-Jboory, 2006 ) |
| Carob moth | *Ectomyelois ceratoniae* Zeller | Algeria, Egypt, Israel, Iraq, Libya,  Morocco, Saudi Arabia, Spain, Tunisia | (Jemni et al., 2014) |
| Dubas bug | *Ommatissus lybicus* de Berg. | Algeria, Bahrain, Egypt, Iraq, Libya, Morocco, Oman, Pakistan, Saudi Arabia, Sudan, Tunisia, Yemen | (Jassim, 2007; Shah et al., 2012) |
| Lesser date moth | *Batrachedra amydraula* Meyrick | Bahrain, Egypt, Iraq, Israel, Kuwait, Libya, Palestine Territories, Saudi Arabia, UAE, Yemen | (Al-Jorany & Al-Delamy, 2010; Levi-Zada et al., 2011; Ba-Angood, 2012) |
| Red palm weevil | *Rhynchophorus ferrugineus* | Bahrain, Egypt, GCC countries, Iraq, Israel, Oman, Pakistan, Qatar, Saudi Arabia, Spain, Syria, Yemen | (Soroker et al., 2005; Al-Asfoor, 2012; Sallam et al., 2012; Abul-Soad, 2013; Al-Habshi, 2014) |
| Red date Scale | *Phoenicococcus marlatti* | Algeria, Egypt, GCC countries, Israel, Jordan, Morocco, Palestine, Spain, Tunisia, | (Gomez, 2002; Lahaam, 2005) |
| Rhinoceros beetle | *Oryctes rhinoceros* L. | Bahrain, Egypt, Iran, Iraq, Oman, Qatar, Tunisia, UAE, Yemen | (Ba-Angood & Al-Baity, 2006) |
| White scale or Parltoria date scale | *Parlatoria blanchardi* | Algeria, Arabia, Bahrain, Iran, Iraq, Kuwait, Libya, Egypt, Mauritania, Morocco, Oman, Pakistan, Palestine, Saudi Arabia, Sudan, Syria, Tunisia, Yemen | (Almansoori et al., 2015) |
| Bastard offshoot | *Batrachedra amydraula* | Bahrain, India, Israel, Pakistan, Palesitine, Syria, Yemen | (Al-Delamy, 2004; Al-Ghurabi & Ba-Angooda, 2001; Al-Jorany & Al-Delamy, 2010; Pezhman, 2005; Levi-Zada et al., 2013) |
| Bunch fading disorder | *Oligonychus afrasiaticus* | Bahrain, Iran, Iraq, Israel, Syria, Yemen | (Ba-Angood & Basshaih, 2000; Pezhman, 2005; Palevsky et al., 2004; Al-Sewidi & Al-Jboory, 2006) |
| Black nose | *Ommatissus binotatus, O. lybicus* | Bahrain, Iran, Iraq, Israel, Oman, Pakistan, Palestine, Syria, Yemen | (Al-Abbasi, 1987; Hassan et al., 2003; Ba-Angood et al., 2009; Shah et al., 2012) |
| Top bending | *Arenipses sabella* | Bahrain, Iran, Iraq, Israel, Spain, Syria | (Rivera et al., 2015) |

**References:**

Abdullah, S.K., Asensio, L., and Monfort, E. (2005) Occurrence in Elx, SE Spain of infl orescence rot disease of date palm caused by Mauginiella scaettae . *J Phytopathol.* 153:1-6

Abul-Soad, A.A. (2011). Date palm in Pakistan, current status and prospective. *Report USAID Pakistan.*

Abul-Soad, A.A. (2013). Current status and perspective of date palm in Pakistan. International conference on date palm: present status & future prospects. Islamia University Bahawalpur, Bahawalpur, 2-3, p 1.

Al-Abbassi, S.H. (1987). Jumping mechanism of dubas bug Ommatissus binotatus de Berg. (Homoptera: Tropiduchidae). *J Agric Water Res.* 6(1):29–38

Al-Asfoor, A.A.A. (2012). A study of some ecological and biological aspects of the red palm weevil in the Kingdom of Bahrain. M.S. Thesis, Arabian Gulf University.

Al-Badran, B.M. (2008). Study the inflorescence rot caused by *Mauginiella scaettae* and *Fusarium* sp and the possibility of chemical and biological control of this disease. MSc thesis, University of Basrah.

Al-Delamy, K.A. (2004). Ecological and economic studies on lesser date moth ( Batrachedra amydraula Meyrick) (*Cosmopterygidae*: *Lepidoptera*) in the middle of Iraq. MSc thesis, College of Agriculture, University of Baghdad.

Al-Ghurabi, A.S., and Ba-Angood, S.A. (2011). Screening of some chemical insecticides for the control of the lesser date moth Batrachedra amydraula Merk on date palm trees at two different sites in the coastal areas of Hadramout, Republic of Yemen. *Univ. Aden. J. Nat. Appl. Sci.* 15(3):565–572

Al-Habshi, K. (2014). The red palm weevil *Rhynchophorus ferrugineus* (Olivier) (*Coleoptera: Curculionidae*). Seiyun Agricultural Research Centre, Seiyun, Yemen

Alhudaib, K., Arocha, Y., Wilson, M., and Jones, P. (2008). First report of a 16SrI, *Candidatus Phytoplasma asteris* Group Phytoplasma associated with a date palm disease in Saudi Arabia. *Plant Pathol.*. 57: 366.

Alhudaib, K., Rezk, A.and Alsalah, M. (2014). Phytoplasma Disease in Date Palm in Saudi Arabia. In: Proceedings of the 5th International Date Palm Conference. Publisher Khalifa International Date Palm Award, United Arab Emirates, Pp. 311-318.

Al-Jorany, R.S., and Al-Delamy, K.A. (2010). Economic losses of lesser date moth ( Batrachedra amydraula Meyrick) (*Cosmopterygidae: Lepidoptera*) on two cultivars, Khastawi and Zahdi of date palm in the middle of Iraq. *Al-Anbar J. Agric. Sci.* 8(2):256–265.

Al-Jorany, R.S., and Al-Delamy, K.A. (2010). Economic losses of lesser date moth (*Batrachedra amydraula* *Meyrick*) (*Cosmopterygidae*: *Lepidoptera*) on two cultivars, Khastawi and Zahdi of date palm in the middle of Iraq. *Al-Anbar J. Agric. Sci.* 8(2):256–265.

AlKhazindar, M. (2014). Detection and molecular identification of aster yellows phytoplasma in date palm in Egypt. *J. Phytopath.* 162 (9): 621-625.

Almnsoori, T.A., Al-Khalifa, M.A., and Mohamed, M.A. (2015). Date palm status and perspectives in Bahrain. In: Date palm genetic resources and utilization. Al-Khayri J.M., Jain S.M. and Johnson D.V. Volume 2: Asia and Europe.

Al-Sakaff, S.M. (2012). Fungal diseases of date palm in Wadi Hadramout. *Yem. J. Agr. Res. Stud.* 26:21–30.

Al-Sewidi, T.M., and Al-Jboory, I.J. (2006). Fecundity of Old World date mite *Oligonychus afrasiaticus* (McGregor) on date palm. *Arab. J. Plant Prot.* 24(1):23–30.

Ammar, M.I., Amer, M.A., and Rashed, M.F. (2005). Detection of phytoplasma associated with yellow streak disease of date palms (*Phoenix dactylifera* L.) in Egypt. *Egypt. J. Virol.* 2:74–86.

Ba-Angood, S.A. (2012). Technologies and methods that could be adopted for the management of insect pests of date palm in Arab countries, with particular reference to Doubas bug. First regional conference on pest management of date palms, Al-Ain, 23–25.

Ba-Angood, S.A., and Al-Baity, S.O. (2006). Monitoring date palm stalk borers *Oryctes* spp using light traps in March 2003- February 2004, and its relationship with different environmental conditions at Seiyun area in Wadi Hadramout- Republic of Yemen. The third international date palm conference, UAE University, Abu Dhabi.

Ba-Angood, S.A., Alghurabi, A., and Hubaishan, M.A. (2009). Biology and chemical control of the old world bug Doubas bug *Ommatissus lybicus* on date palm trees in the coastal areas of Hadramout Governorate. Republic of Yemen. *Arab. J. Plant Prot.* 27:1–9.

Ba-Angood, S.A., and Basshaih, G.S. (2000). A study on the effect of date palm dust mite *Oligonychus afrasiaticus* (McGregor) (*Acarin*: *Tetranychidae*) on the physiochemical characters of three different date varieties in Wadi Hadhramout. *Yemen Arab. J. Plant Prot.* 18(2):82–85.

Gómez, S. (2002). Cría masiva de Rhyzobius lophanthae Blaisdell (Coleoptera: Coccinellidae) depredador de la cochinilla roja de las palmeras ( Phoenicococcus marlatti Cockerell). *Bol. San. Veg. Plagas.* 28:167–176.

Gurr, G. M., Bertaccini, A., Gopurenko, D., KruegerR.R., Alhudaib, K.A., Liu, J., Fletcher, M.J., et al. (2015). Phytoplasmas and thier insect vectors: Implications for date palm. In Wakil, W., Falero,J.R., and Miller T. A. (Eds.), Sustainable pest management in date palm: Current status and emerging challenges (pp. 287–314). Cham: Springer.

Harrison, N.A., and Elliott, M.L. (2016). Phytoplasmas associated with date palm in the continental USA: three 16SrIV subgroups. *Emirates J. Food Agric.* 28 (1) : 17-23.

Hasan, B.H., Al-Jboory, I., Al-Rubeai, H., Viggiani, G., et al. (2003). *Pseudoligosita* *babylonica* n. sp. (*Hymenoptera*: *Trichogrammatidae*), egg parasitoid of *Ommatissus* *lybicus* Berg. (*Homoptera*: *Tropiduchidae*) in Iraq. *Boll. Lab. Ent. Agr. Filip. Silv.* 59:75-78.

Jassim, H.K. (2007). Studies on the biology of palm dubas bug *Ommatissus* *lybicus* (*Debergevin*.) Asche and Wilson. (*Homoptera: Tropiduchidae*) and its biocontrol by some isolates of entomopathogenic fungi *Beauveria* *bassiana* (Balsamo) Vuill. and Lecanicillium (*Verticillium*) lecanii (Zimm.) Zare and Oami. PhD thesis, College of Agriculture, University of Baghdad, Bagdad, Iraq

Jemni, M., Otón, M., and Ramirez, J.G. (2014). Conventional and emergent sanitizers decreased Ectomyelois *ceratoniae* infestation and maintained quality of date palm after shelf-life. *Post Harv Biol. Tech*. 87:33-41.

Lahaam, S. (2005). Date palm in Jericho. Report, Ministry of Agriculture, Palestine.

Levi-Zada, A., Fefer, D., and Anshelevitch, L. (2011). Identification of the sex pheromone of the lesser date moth, *Batrachedra amydraula* , using sequential SPME auto-sampling. *Tetrahedron Let*t. 52(35):4550–4553.

Levi-Zada, A., Sadowsky, A., and Dobrinin, S. (2013). Reevaluation of the sex pheromone of the lesser date moth, *Batrachedra amydraula* , using autosampling SPME-GC/MS and field bioassays. Chemoecology. 2(1):13–20.

Palevsky, E., Ucko, O., Peles, S. (2004). Evaluation of control measures for Oligonychus afrasiaticus infesting date palm cultivars in the Southern Arava Valley of Israel. *Crop Prot.* 23(5):387–392.

Palevsky, E., Ucko, O., and Peles, S. (2004). Evaluation of control measures for *Oligonychus* *afrasiaticus* infesting date palm cultivars in the Southern Arava Valley of Israel. *Crop Prot* 23(5):387–392

Rivera, D., Obón, C., Alcaraz, F., Carreño, E., Laguna, E., Asmoros, A., Johnson, D.V., Diaz, G., Morte, A., et al. (2015). Date Palm Status and Perspective in Spain In: Date Palm Genetic Resources and Utilization. Al-Khayri, J.M., Jain, S.M. and Johnson, D.V.. Volume 2: Asia and Europe. Springer USA.

Saeed, E.E., Sham, A., E-Tarabily, K., Elsamen, F.A., Iratni, R., AbuQamar, S.F., et al. (2016). Chemical control of black scorch disease on date palm caused by the fungal pathogen *Thielaviopsis* *punctulata* in United Arab Emirates*. Plant Dis.* 100 (12): 2370-2376. doi.org/10.1094/PDIS-05-16-0645-RE

Sallam, A.A., El-Shafi, H.A.F., and Al-Abdan, S. (2012). Influence of farming practices on infestation by red palm weevil *Rhynchophorus* *ferrugineus* (Olivier) in date palm: a case study. *Int. Res. J. Agric. Sci. Soil Sci.* 2(8):370–376.

Shah, A., Mohsin, A., and Naeem, M. (2012) Biology of Dubas Bug, *Ommatissus* *lybicus* (*Homoptera*: *Tropiduchidae*), a pest on date palm during spring and summer seasons in Panjgur, Pakistan. *Pak. J. Zool.* 44(6):1603–1611.

Soroker, V., Blumberg, D., Haberman, A., Hamburger-Rishard, M., et al. (2005). Current status of red palm weevil infestation in date palm plantations in Israel. *Phytoparasitica.* 33(1):97–106.
